# Long noncoding RNAs as tumorigenic factors and therapeutic targets for renal cell carcinoma

**DOI:** 10.1186/s12935-021-01805-2

**Published:** 2021-02-16

**Authors:** Haiyan Shen, Guomin Luo, Qingjuan Chen

**Affiliations:** 1 Department of Nephrology, 3201 Hospital, Hanzhong, Shaanxi Province China; 2 Department of Oncology, Yongchuan Hospital of Chongqing Medical University, Chongqing, 40016 China

**Keywords:** Long noncoding RNA, Gene expression, Renal cell carcinoma, Tumor initiation, Tumor progression, Prognosis

## Abstract

Approximately 338,000 patients are diagnosed with kidney cancer worldwide each year, and renal cell carcinoma (RCC), which is derived from renal epithelium, accounts for more than ninety percent of the malignancy. Next generation RNA sequencing has enabled the identification of novel long noncoding RNAs (lncRNAs) in the past 10 years. Recent studies have provided extensive evidence that lncRNAs bind to chromatin modification proteins, transcription factors, RNA-binding proteins and microRNAs, and thereby modulate gene expression through regulating chromatin status, gene transcription, pre-mRNA splicing, mRNA decay and stability, protein translation and stability. In vitro and in vivo studies have demonstrated that over-expression of oncogenic lncRNAs and silencing of tumor suppressive lncRNAs are a common feature of human RCC, and that aberrant lncRNA expression is a marker for poor patient prognosis, and is essential for the initiation and progression of RCC. Because lncRNAs, compared with mRNAs, are expressed in a tissue-specific manner, aberrantly expressed lncRNAs can be better targeted for the treatment of RCC through screening small molecule compounds which block the interaction between lncRNAs and their binding proteins or microRNAs.

## Introduction

Long non-coding RNAs (lncRNAs) are RNAs more than 200 nucleotides in length without protein-coding capacity, but resemble mRNAs as they are also transcribed by RNA polymerase II and are subjected to post-transcriptional capping, splicing and polyadenylation [1, 2]. LncRNAs originate from the transformation of protein-coding genes through mechanisms such as new promoters; non-transcribed regions after chromosomal rearrangements; noncoding gene duplication; and non-transcribed regions with transposable element sequences [3, 4].

By analyzing comprehensive systems biology-generated datasets, St. Laurent et al. have proposed lncRNA classification based on ten aspects, including lncRNA length, mRNA resemblance, sequence and structure conversation, subcellular structures, association with annotated protein-coding genes or other known DNA elements, biochemical pathway or stability, biological states and functions [5]. More recently, Kopp and Mendell have proposed to classify lncRNAs according to their functions: lncRNAs can be classified into those that act *in cis*, regulating chromatin state and/or the expression of nearby genes, and those that exert a range of functions throughout the cell *in trans* [2].

Recent studies have shown that a number of oncogenic lncRNAs are significantly over-expressed while a number of tumor suppressive lncRNAs are considerably down-regulated in human renal cell carcinoma (RCC) tissues, and that the aberrant lncRNA expression is a marker for poor patient prognosis and is essential for the initiation and progression of RCC [6–11]. Through binding to proteins and microRNAs (miRNAs), lncRNAs play important roles in regulating gene transcription, pos-transcriptional mRNA expression, protein synthesis, post-translational protein stability, RCC cell proliferation, survival, migration, invasion, tumor initiation and progression [6, 7, 9–13]. The present review focuses on the regulation of gene and protein expression by lncRNAs, lncRNAs as oncogenes or tumor suppressors for RCC, and lncRNAs as targets for RCC therapy.

## Regulation of gene and protein expression by lncRNAs

LncRNAs have emerged as key regulators of gene expression at transcriptional and post-transcriptional levels, including chromatin modification, gene transcription [14, 15], pre-mRNA splicing [16], RNA stabilization or decay [17, 18], protein translation [19], stabilization and degradation [20].

### LncRNAs regulate gene transcription *in cis* [2, 21]

The transcription of lncRNAs can alter RNA polymerase II occupancy at the promoters and gene bodies of their neighboring protein-coding genes through modulating transcription factor binding on their promoter and enhancer regions and chromatin status [2, 21–23]. For example, during embryonic stem cell differentiation, the lncRNA Haunt directly represses HOXA gene enhancers and reduces HOXA gene expression by direct negative regulation of *cis*-regulatory elements [24].

Depletion of a number of lncRNAs results in reduction in the expression of their neighboring protein-coding genes, such as the master regulators of hematopoiesis TAL1, Snai1 and Snai2 [25]. Heterologous transcription assays demonstrate that the lncRNAs are required for the expression of their neighboring protein-coding genes [25]. Mechanistically, lncRNAs transcribed from gene enhancer or non-enhancer regions can show enhancer-like functions. For example, the lncRNA ncRNA-a forms looping with its target gene DNA, recruits Mediator to ncRNA-a target genes, regulates chromatin localization and kinase activity of Mediator, leading to transcriptional activation of neighboring protein-coding genes [14].

### LncRNAs regulate gene transcription *in trans*

Polycomb repressive complex 2 (PRC2), including its catalytic histone methyltransferase subunit EZH2, plays an important role in lncRNA-regulated transcriptional repression [26, 27]. The lncRNA HOTAIR recruits PRC2 to the HOXD gene locus, leading to histone H3 lysine 27 trimethylation (H3K27me3) and transcriptional repression of the HOXD gene [26]. The lncRNA HERES interacts with EZH2 via its G-quadruple structure-like motif to repress CACNA2D3, SFRP2 and CXXC4 expression simultaneously, leading to Wnt signaling pathway activation and esophageal squamous cell carcinoma cell proliferation, migration, invasion, colony formation and tumor growth [28].

**Fig. 1:**
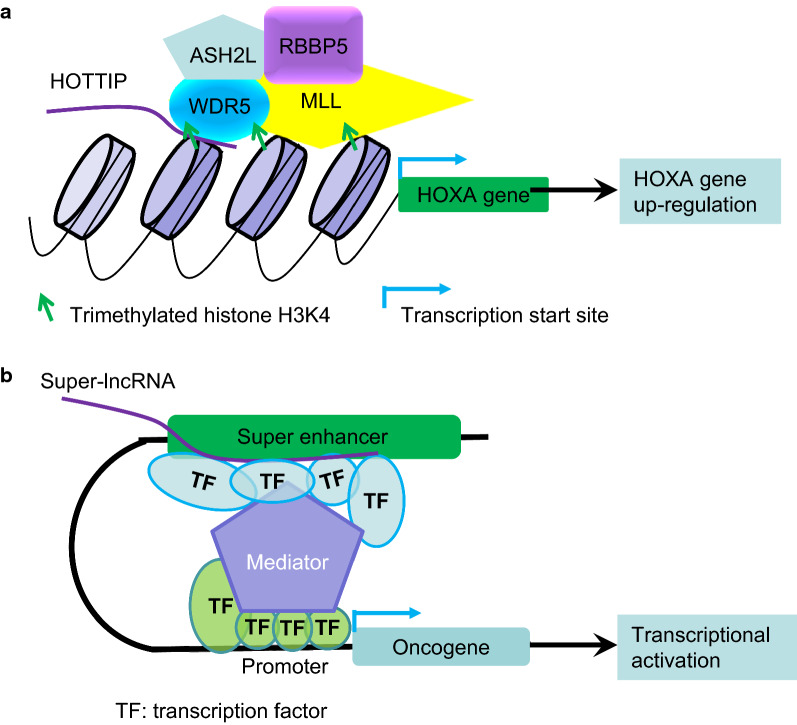
LncRNAs regulate gene transcription. **a** The lncRNA TOTTIP forms a RNA–protein complex with WDR5 and recruits the histone H3 lysine 27 trimethylation MLL/WDR5/ASH2L/RBBP5 protein complex to the HOXA gene promoter, leading to histone H3 lysine 4 (H3K4) trimethylation and transcriptional activation of the HOXA gene. **b** Super-lncRNAs bind to multiple anchor DNA sites at super-enhancers, and recruit and transport transcription factors (TFs) and Mediator complexes to super-enhancers, leading to transcriptional activation of super-enhancer-associated oncogenes

LncRNAs are now well-known to activate target gene transcription through interacting with histone modification proteins, transcriptional super-enhancers or Mediator. WDR5, a component of the MLL histone methyltransferase complex, promotes histone H3 lysine 4 (H3K4) trimethylation and transcriptional activation [29]. The lncRNA HOTTIP binds to WDR5 and thereby recruits the WDR5-MLL histone H3K4 methyltransferase complex to the HOXA gene locus to induce HOXA gene transcription (Fig. 1a) [30]. The lncRNA GClnc1, which is up-regulated in human gastric cancer tissues, binds to WDR5 and the histone acetyltransferase KAT2A, acts as a modular scaffold for WDR5 and KAT2A complexes, and promotes the transcription of their target genes including SOD2, leading to gastric cancer cell proliferation, invasion and metastasis [15]. Super-enhancers are characterized by binding of abundant transcription factors and the BET bromodomain protein BRD4 and are associated with high levels of Mediator [31, 32]. A subset of lncRNAs, which are termed super-lncRNAs, form RNA and DNA complexes with multiple anchor DNA sites within the super-enhancers, and super-lncRNAs in a particular cluster share common short structural motifs which recruit and transport transcription factors and Mediator complexes to super-enhancers [33]. Super-lncRNAs therefore play a critical role in chromatin organization and transcriptional activation of super-enhancer-associated oncogenes (Fig. 1b) [33]. In addition, RNA exosomes protects divergently transcribed lncRNA expressing enhancers by resolving deleterious transcription-coupled secondary DNA structures, while also regulating long-range super-enhancer chromosomal interactions important for cellular function [33].

### LncRNAs regulate alternative splicing, mRNA stability and decay

Antisense lncRNAs and sense mRNAs form complementary base pairs to cover splicing sites, thereby regulating mRNA splicing. For example, the antisense lncRNA NAT binds to the 5′ splicing site of Zeb2 mRNA, blocking Zeb2 5′ untranslated region intron editing and thereby inducing Zeb2 mRNA translation. NAT overexpression therefore suppresses Zeb2 pre-mRNA splicing and enhances Zeb2 mRNA translation [16]. The lncRNA EPR interacts with chromatin, up-regulates p21 gene transcription by interacting with SMAD3, and down-regulates p21 mRNA decay by interacting with the mRNA decay-promoting factor KHSRP, leading to p21 mRNA up-regulation and growth inhibition of tumor cells and tumors [34]. In triple negative breast cancer cells, ~ 500 natural antisense transcript (NAT) lncRNAs are deregulated and show co-expression with oncogenic and tumor suppressor protein-coding genes *in cis* [35]. The NAT lncRNA PDCD4-AS1 forms duplex with PDCD4 mRNA, controls the interaction between PDCD4 mRNA and RNA decay promoting factors including HuR, enhances PDCD4 mRNA stability and thereby positively regulates the expression and activity of the tumor suppressor PDCD4 [35]. In addition, both PDCD4-AS1 and PDCD4 are down-regulated in triple negative breast cancer cell lines and human tumor tissues, and the tumorigenic properties of PDCD4-AS1-depleted tumor cells can be rescued by forced PDCD4 over-expression [35].

### LncRNAs regulate protein translation and stability

The lncRNA lncNB1 directly binds to the ribosomal protein RPL35 and promotes the translation of tumorigenic mRNAs such as E2F1, leading to transcriptional activation of E2F1 target genes such as DEPDC1B, N-Myc protein phosphorylation at serine 62 and stabilization and neuroblastoma tumorigenesis [19]. The antisense lncRNA PU.1 AS is transcribed from an intronic promoter of the PU.1 protein-coding gene. In the cytoplasm, PU.1 AS binds to PU.1 mRNA, forming a RNA complex and thereby suppressing PU.1 mRNA translation [36]. The lncRNA lncRNA-p21 is transcriptionally activated by hypoxia-inducible factor 1α (HIF1α) under hypoxic conditions and contributes to hypoxia-induced glycolysis. In a feedback loop, lncRNA-p21 binds to HIF1α and von Hippel-Lindau (VHL) proteins and prevents VHL-mediated HIF1α protein ubiquitination, leading to HIF1α protein stabilization and over-expression [37].

## LncRNAs in renal cell carcinoma

Approximately 338,000 patients are diagnosed with kidney cancer worldwide each year, and renal cell carcinoma (RCC), which is derived from renal epithelium, accounts for more than ninety percent of the malignancy [38]. As lncRNAs are involved in all aspects of gene expression regulation, including gene transcription, pre-mRNA splicing, RNA stability and decay, protein translation and stability, it is not surprising that lncRNAs play an important role in tumor initiation and progression. Accumulating evidences demonstrate that lncRNAs are dysregulated in human RCC tissues and play critical roles in RCC tumorigenesis.

## LncRNAs as oncogenes for renal cell carcinoma

### ANRIL

Antisense lncRNAs regulate the expression of their neighboring protein coding genes to promote tumorigenesis. The INK4b-ARF-INK4a gene locus encodes the cyclin-dependent kinase inhibitors p15INK4b and p16INK4a and the p53 tumor suppressor regulator ARF. The antisense strand of the INK4b-ARF-INK4a locus transcribes the antisense lncRNA ANRIL, which interacts with chromatin modification protein CBX7 to epigenetically silence the tumor suppressor INK4a [39]. ANRIL is highly expressed in human RCC tissues and RCC cell lines, and significantly promotes RCC cell proliferation, migration, invasion and epithelial-to-mesenchymal transition (EMT) [6] (Table 1).

**Table 1:** Oncogenic lncRNAs in renal cell carcinoma

LncRNAs	Functions	References
ANRIL	Is over-expressed in RCC tissues; interacts with CBX7 to suppress INK4a expression; promotes RCC cell proliferation, migration, invasion, and epithelial-to-mesenchymal transition (EMT)	[6, 39]
EGFR-AS1	Is upregulated in human RCC tissues and predicts poor patient prognosis; binds to HuR protein and EGFR mRNA to suppress EGFR mRNA degradation; induces RCC cell proliferation and invasion	[7]
HOTAIR	Is over-expressed in RCC tissues and predicts poor patient prognosis; up-regulates insulin growth factor-binding protein 2; competes with miR-217 to up-regulate HIF1α and AXL; induces RCC cell proliferation, migration and EMT in vitro, tumorigenicity and lung metastasis in vivo	[8, 40]
MALAT1	Is expressed at higher levels in RCC tissues and predicts poorer patient prognosis; interacts with EZH2 to suppress E-cadherin and up-regulate β-catenin; induces RCC cell proliferation, invasion and metastasis	[9]
PCAT1	Is over-expressed in human RCC tissues; acts as a sponge for miR-656 and miR-539 to up-regulate YAP; induces RCC cell proliferation, migration and invasion	[46]
H19	Is over-expressed in human RCC tissues and associated with poorer patient prognosis; binds miR-29a-3p to suppress E2F1 regulation; induces RCC cell proliferation, invasion and migration	[48, 49]
SNHG12	Is highly expressed in RCC tissues and predicts poorer patient prognosis; binds to SP1 and blocks its degradation; enhances CDCA3 gene transcription; induces RCC cell proliferation, migration, invasion, tumor growth in vivo and resistance to sunitinib treatment	[51–53]
LINC00152	Is up-regulated in human RCC tissues and predicts poor patient survival; binds to miR-205, EZH2 and LSD1 proteins; induces p16 gene suppression, leading to RCC cell proliferation	[54]
LINC01094	Is over-expressed in human RCC tissues; functions as a sponge of miR-224-5p to regulate CHSY1 expression; induces RCC cell proliferation, invasion, tumor growth and metastasis	[55]
LncARSR	Is upregulated in primary renal tumor-initiating cells and associates with poor patient prognosis; induce YAP protein nuclear translocation; promotes tumor-initiating cell renewal, tumorigenicity and metastasis	[56]

### EGFR-AS1

The antisense lncRNA EGFR-AS1 (epidermal growth factor receptor antisense RNA 1) is transcribed from the opposite strand of the oncogene EGFR, directly binds to EGFR mRNA and suppresses its degradation [7]. RNA-binding protein pull-down and mass spectrometry analyses have identifies HuR, which is responsible for EGFR mRNA stability, as an EGFR-AS1 binding protein [7]. Gain- and loss-of-function studies demonstrate that EGFR-AS1 induces RCC cell proliferation and invasion in vitro and in mice. In human RCC tissues, EGFR-AS1 is significantly up-regulated, and higher EGFR-AS1 expression predicts a poor patient prognosis, independent of other prognostic markers [7]. EGFR-AS1 therefore promotes RCC through interacting with HuR to up-regulate EGFR expression, and is a prognostic biomarker and a therapeutic target for RCC (Table 1).

### HOTAIR

The antisense lncRNA HOTAIR (HOX transcript antisense RNA) is transcribed from a regulatory boundary of the HOXC gene locus. HOTAIR binds the polycomb repressive PRC2 complex as well as the histone demethylase LSD1/CoREST/REST complex at the HOXD gene locus, resulting in transcriptional repression of anti-metastasis genes and breast cancer metastasis [12, 13]. In human RCC tissues, HOTAIR expression is over-expressed, and HOTAIR over-expression in tumor tissues is correlated with tumor progression and poor patient prognosis [8]. Gain- and loss-of-function studies reveal that HOTAIR up-regulates insulin growth factor-binding protein 2 expression and also functions as a competing endogenous RNA for miR-217 to enhance HIF1α and AXL expression, leading to RCC cell proliferation, migration and EMT [8, 40]. Additionally, HOTAIR knockdown inhibits tumor growth and HOTAIR overexpression accelerates tumorigenicity and lung metastasis in mouse models of RCC [8, 40] (Table 1).

### MALAT1

MALAT1 (metastasis-associated in lung adenocarcinoma transcript 1) is predominantly found in nuclear speckles [41]. While MALAT1 has been shown to promote cancer cell migration, invasion and metastasis in a variety of cancer types [42, 43], MALAT1 is expressed at higher levels in human RCC tissues than normal tissues, and higher levels of MALAT1 expression in human RCC tissues are associated with poorer patient prognosis [9]. Mechanistically, MALAT1 interacts with EZH2 protein to suppress the expression of E-cadherin, up-regulate the expression of β-catenin and induce epithelial-mesenchymal transition, and MALAT1 silencing reduces RCC cell proliferation, invasion and metastasis [9]. However, MALAT1 has recently been reported to block TEAD from association with its co-activator YAP and target gene promoters, and to suppress breast cancer metastasis in transgenic, xenograft and syngeneic mouse models [44] (Table 1).

### PCAT1

The lncRNA PCAT1 (prostate cancer associated transcript 1) gene is localized upstream of the MYC oncogene, and was first found to be considerably over-expressed in a subset of human metastatic prostate cancer tissues [45]. PCAT1 levels are significantly higher in human RCC tissues, and PCAT1 knockdown reduces RCC cell proliferation, migration and invasion [46]. Mechanistically, PCAT1 up-regulates the expression of YAP protein by acting as a sponge for miR-656 and miR-539 [46] (Table 1).

### H19

The lncRNA H19 is transcribed only from the maternal allele [47]. H19 expression is significantly higher in human RCC tissues, compared with adjacent normal renal tissues, and higher levels of H19 in RCC tissues are associated with poorer patient prognosis, independent of other prognostic markers [48]. H19 competitively binds to miR-29a-3p to suppress miR-29a-3p-mediated E2F1 regulation, leading to RCC cell proliferation, invasion and migration [48, 49] (Table 1).

### SNHG12

The lncRNA SNHG12 (small nucleolar RNA host gene 12) has been shown to play a key role in tumor initiation and progression in different organs through a variety of mechanisms [50]. SNHG12 is highly expressed in RCC tissues and in sunitinib-resistant RCC cells, and higher levels of SNHG12 in human RCC tissues are associated with poorer patient prognosis [51–53]. SNHG12 binds to the transcription factor SP1 and blocks its ubiquitylation and degradation, and SP1 directly binds to the CDCA3 gene promoter and enhances its transcription, leading to RCC cell proliferation, migration, invasion and resistance to sunitinib treatment. In addition, SNHG12 knockdown reverses RCC tumor resistance to sunitinib treatment and SNHG12 induces tumor growth in mouse models [51] (Table 1).

### LINC00152

The lncRNA LINC00152 is significantly up-regulated in human RCC tissues compared with adjacent normal tissues, and a high level of LINC00152 expression is positively correlated with lymph node metastasis, advanced disease stage, and poor over survival in RCC patients [54]. LINC00152 binds to the chromatin modifying EZH2 and LSD1 proteins and induces histone H3 lysine 27 tri-methylation at the p16 gene promoter and p16 gene transcriptional suppression, leading to RCC cell proliferation [54]. In addition, miR-205 is a target of LINC00152, and LINC00152 represses miR-205 function [54]. LINC00152 is therefore likely to contribute to RCC tumorigenesis by epigenetically inhibiting p16 gene expression and interacting with miR-205 (Table 1).

### LINC01094

Analysis of The Cancer Genome Atlas datasets show that the lncRNA LINC01094 is highly expressed in human RCC tissues [55]. Transcriptionally activated by the oncogenic transcription factor FOXM1, LINC01094 functions as a sponge of miR-224-5p and regulates CHSY1 expression via competitively binding to miR-224-5p, leading to RCC cell proliferation and invasion in vitro and tumor growth and metastasis in a mouse model [55]. LINC01094 therefore exerts oncogenic effects in RCC through the miR-224-5p/CHSY1 regulatory axis [55] (Table 1).

### LncARSR

Renal tumor-initiating cells play a critical role in RCC tumor initiation, progression and drug resistance. The lncRNA lncARSR is up-regulated in primary renal tumor-initiating cells and associated with poor prognosis in RCC patients [56]. LncARSR knockdown suppresses renal tumor-initiating cell self-renewal, tumorigenicity and metastasis, and forced lncARSR over-expression enhances RCC cell tumor-initiating function [56]. Mechanistically, in a positive feedback loop, lncARSR gene transcription is activated by YAP protein, and lncARSR binds to YAP to repress LATS1-mediated YAP protein phosphorylation and induce YAP nuclear translocation [56]. LncARSR therefore plays a critical role in renal tumor-initiating cell propagation and serves as a biomarker for poor patient prognosis and as a therapeutic target [56] (Table 1).

## LncRNAs as tumor suppressors for renal cell carcinoma

### MEG3

The lncRNA MEG3 is known to function as a tumor suppressor in glioma by reducing MDM2 gene expression, leading to p53 tumor suppressor protein stabilization and over-expression, and glioma cell growth arrest and programmed cell death [57]. Analysis of The Cancer Genome Atlas datasets reveals decreased MEG3 expression in human RCC tissues [10]. MEG3 up-regulates RASL11B expression by binding to, competing with and suppressing miR-7, leading to reduction in RCC cell proliferation, migration and invasion, and induction in RCC cell cycle arrest and apoptosis [10] (Table 2).

**Table 2:** Tumor suppressive lncRNAs in renal cell carcinoma

lncRNAs	Functions	References
MEG3	Is decreased in human RCC tissues; up-regulates RASL11B expression by competing with miR-7; reduces RCC cell proliferation, migration and invasion and induces RCC cell apoptosis	[10]
lncPENG	Is expressed at lower levels in human RCC tissues with the down-regulation correlating with worse patient prognosis; binds to miR-15b to up-regulate PDZK1 expression; suppresses RCC cell proliferation in vitro and in vivo	[11]
lncRNA-LET	Is expressed at lower levels in human RCC tissues; competes with miR-373-3p to block DKK1 and TIMP2 down-regulation; induces RCC cell cycle arrest and apoptosis; blocks RCC tumor growth in mice	[58]
KCNQ1DN	Is down-regulated in human RCC tissues; reduces c-Myc and cyclin D1 and up-regulates p27; suppresses RCC cell proliferation and cell cycle progression; suppresses RCC tumor growth	[59]
OTUD6B-AS1	Is down-regulated in human RCC tissues with the down-regulation correlating with poor patient prognosis; blocks Wnt/β-catenin pathway, reduces EMT-related proteins; suppresses RCC cell proliferation, survival, migration and invasion in vitro and tumor growth in mice	[60]
LncRNA-SARCC	Is silenced in human RCC tissues with the down-regulation predicting worse patient prognosis; destabilizes androgen receptor; de-represses miR-143-3p; inactivates ERK, AKT, MMP-13 and K-RAS; reduces RCC cell proliferation, migration and invasion in vitro and in mice	[61, 62]
FILNC1	Is down-regulated in RCC tissues with the down-regulation correlating with worse patient prognosis; downregulates c-Myc protein translation; reduces glucose uptake and lactate production; induces energy stress-induced apoptosis and suppresses RCC progression in vivo	[63]

### LncPENG

The lncRNA lncPENG directly binds to miR-15b and effectively acts as a sponge of miR-15b to modulate the expression of PDZK1 [11], which inhibits RCC initiation and progression. MiR-15b knockdown or forced lncPENG over-expression significantly up-regulates PDZK1 expression and suppresses RCC cell proliferation in vitro and in a mouse model [11]. In addition, lncPENG is expressed at lower levels in human RCC tissues, and its down-regulation is associated with a lower PDZK1 level and a higher miR-15b level, larger tumor size and poorer patient prognosis [11]. LncPENG therefore functions as a competing endogenous RNA to suppress miR-15b-mediated PDZK1 downregulation and RCC (Table 2).

### LncRNA-LET

The lncRNA lncRNA-LET (lncRNA-low expression in tumor) acts as a competing endogenous RNA of the oncomiR miR-373-3p, and blocks miR-373-3p-mediated Dickkopf-1 (DKK1) and tissue inhibitor of metalloproteinase-2 (TIMP2) down-regulation [58]. Forced lncRNA-LET overexpression induces RCC cell cycle arrest, impairs mitochondrial membrane potential and induces apoptosis, whereas lncRNA-LET knockdown shows the opposite effects [58]. Importantly, lncRNA-LET suppresses RCC tumor growth in mice, and lncRNA-LET is expressed at lower levels, while miR-373-3p is expressed at higher levels, in human RCC tissues than matched adjacent non-tumor tissues [58] (Table 2).

### KCNQ1DN

The lncRNA KCNQ1DN (KCNQ1 downstream neighbor) is significantly decreased in human RCC tissues, relative to adjacent normal tissues, due to KCNQ1DN gene proximal promoter hypermethylation [59]. KCNQ1DN suppresses RCC cell proliferation and cell cycle progression by inhibiting the transcription and reducing the expression of c-Myc, which up-regulates cyclin D1 and down-regulates p27 [59]. In mouse models of RCC, KCNQ1DN overexpression substantially suppresses the growth of RCC tumors, which correlates with a reduction in c-Myc expression [59]. KCNQ1DN therefore suppresses RCC in vitro and in vivo through repressing c-Myc oncogene transcription and expression, and KCNQ1DN down-regulation is a marker for poor patient prognosis and a therapeutic target (Table 2).

### OTUD6B-AS1

The lncRNA OTUD6B-AS1 (OTUD6B antisense RNA 1) is down-regulated in human RCC tissues, and lower OTUD6B-AS1 expression in RCC tissues correlates with poor patient prognosis [60]. OTUD6B-AS1 suppresses RCC cell proliferation, induces apoptosis, inhibits cell migration and invasion, and significantly represses tumor growth in mice [60]. Mechanistically, OTUD6B-AS1 inactivates the Wnt/β-catenin pathway and suppresses the expression of epithelial-to-mesenchymal transition-related proteins, including E-cadherin, N-cadherin and Snail in RCC cells [60]. The lncRNA OTUD6B-AS1 therefore exerts tumor suppressive effects by inactivating the Wnt/β-catenin pathway (Table 2).

### LncRNA-SARCC

The gene promoter of the lncRNA lncRNA-SARCC (lncRNA-suppressing androgen receptor in renal cell carcinoma) is highly methylated in human RCC tissues compared with counterpart normal renal tissues, leading to gene silencing, and lower levels of lncRNA-SARCC expression in RCC tissues predict worse prognosis in patients [61, 62]. LncRNA-SARCC binds to and destabilizes androgen receptor protein, and thereby transcriptionally de-represses miR-143-3p expression, leading to inactivation of ERK, AKT, MMP-13 and K-RAS. LncRNA-SARCC thus reduces RCC cell proliferation, migration and invasion in vitro and in a mouse model [62] (Table 2).

### FILNC1

The lncRNA FILNC1 (FoxO-induced long noncoding RNA 1) is expressed at higher levels in kidney than other organs. FILNC1 is down-regulated in RCC tissues, compared with corresponding normal tissues, and a low level of FILNC1 in RCC tissues correlates with poor patient prognosis [63]. Upon energy stress, FILNC1 is up-regulated by the transcription factor FoxO, interacts with the c-Myc mRNA-binding protein AUF1, sequesters AUF1 from c-Myc mRNA, and thereby downregulates c-Myc protein translation [63]. FILNC1 knockdown in RCC cells enhances glucose uptake and lactate production through up-regulating c-Myc, suppresses energy stress-induced apoptosis and promotes RCC progression in vivo [63]. FILNC1 is therefore a negative regulator of RCC, and FILNC1 down-regulation is a marker for poor patient prognosis and a valid therapeutic target [63] (Table 2).

## LncRNAs as targets for anticancer therapy

Because tumorigenic lncRNAs are over-expressed and tumor suppressive lncRNAs are down-regulated in human RCC tissues and the dysregulation correlates with poor patient prognosis, aberrant lncRNA expression has been proposed as valuable biomarkers for patient prognosis and diagnosis. As lncRNAs are often expressed in a strikingly tissue-specific manner, compared with mRNAs [64], lncRNAs are desirable targets for cancer therapy, and lncRNA-targeting anticancer agents are currently under development.

Antisense oligonucleotide (ASO) technologies and nanoparticle-mediated RNA interference can be used to knock down oncogenic lncRNAs that are overexpressed in cancers, and ASOs and nanoparticle-mediated RNA interference targeting lncRNAs have shown promising anticancer effects. For instance, the antisense lncRNA WISP1-AS1 overlaps with the fourth intron and fifth exon of the WISP1 gene, and is expressed at higher levels in RCC cell lines and tissues compared with primary proximal tubule cells and adjacent normal tissues from the same patient [65]. Treatment with locked nucleic acid (LNA) gapmeR ASO targeting WISP1-AS1 regulates Egr-1 and E2F gene transcription and results in RCC cell apoptosis [65]. In comparison, nanoparticle-mediated RNA interference of oncogenic lncRNAs has shown promising anticancer effects in mouse models of other cancers [66] but has not been tested in RCC models.

No small molecule compounds targeting lncRNAs are available today for cancer therapy. One way to block the oncogenic effects of lncRNAs is to block their gene transcription. For example, the oncogenic lncRNA lncTASR is transcriptionally up-regulated by TR4, increases AXL protein expression via enhancing AXL mRNA stability, and thereby induce RCC resistance to sunitinib treatment [67]. Inhibition of TR4 with small molecule compounds, such as tretinoin, blocks lncTASR gene transcription, leading to sensitivity of RCC cells to sunitinib therapy in vitro and suppression of RCC tumor progression in mouse models [67].

The most promising way to target lncRNAs is probably by identifying small molecule compounds which block the interaction between lncRNAs and their binding proteins. While this approach is still in its infancy, AlphaScreen (Amplified luminescent proximity homogeneous assay Screen) technology has been shown to effectively quantify the interaction between lncRNAs and their binding proteins for high-throughput screening of small molecule compound libraries [68].

## Conclusions

Advances in RNA sequencing technology has led to the discovery of a large number of novel lncRNAs in the past decade, and lncRNAs are now classified mainly according to their genomic location relative to their neighboring protein coding genes and biological functions. LncRNAs bind to chromatin modification proteins, transcription factors, RNA-binding proteins and miRNAs, and thereby modulate gene and protein expression through regulating chromatin status *in cis* and *in trans*, transcription of their target genes, pre-mRNA splicing, mRNA decay and stability, protein translation, stability and function.

In human RCC tissues, a number of tumorigenic lncRNAs are up-regulated and tumor suppressive lncRNAs are repressed, and the aberrant lncRNA expression is a marker for poor patient prognosis. In vitro and in vivo studies have demonstrated that over-expression of oncogenic lncRNAs and silencing of tumor suppressive lncRNAs result in renal tumor-initiating cell self-renewal, RCC cell proliferation, migration and invasion in vitro and tumor progression in mouse models. Because lncRNAs, compared with mRNAs, are expressed in a tissue-specific manner, aberrantly expressed lncRNAs can be targeted for the treatment of RCC through antisense oligonucleotides and through screening small molecule compounds for agents which block the interaction between lncRNAs and their binding proteins or miRNAs.

## Data Availability

Not applicable.

## Abbreviations

DKK1: Dickkopf-1
EGFR-AS1: Epidermal growth factor receptor antisense RNA 1
EMT: Epithelial-to-mesenchymal transition
FILNC1: FoxO-induced long noncoding RNA 1
H3K4: Histone H3 lysine 4
H3K27me3: Histone H3 lysine 27 trimethylation
HOTAIR: HOX transcript antisense RNA
KCNQ1DN: KCNQ1 downstream neighbor
LNA: Locked nucleic acid
lncRNAs: Long noncoding RNAs
lncRNA-LET: LncRNA-low expression in tumor
lncRNA-SARCC: LncRNA-suppressing androgen receptor in renal cell carcinoma
MALAT1: Metastasis-associated in lung adenocarcinoma transcript 1
NAT: Natural antisense transcript
OTUD6B-AS1: OTUD6B antisense RNA 1
PCAT1: Prostate cancer associated transcript 1
PRC2: Polycomb repressive complex 2
RCC: Renal cell carcinoma
SNHG12: Small nucleolar RNA host gene 12
TIMP2: Tissue inhibitor of metalloproteinase-2
VHL: Von Hippel-Lindau
